# Complete Testicular-Epididymal Non-fusion in an Undescended Testis: Anatomical Features of a Diagnostic Challenge With Clinical Significance

**DOI:** 10.7759/cureus.95887

**Published:** 2025-11-01

**Authors:** Ierotheos Loukas, Dimosthenis Chrysikos, Theodore Troupis

**Affiliations:** 1 Department of Anatomy, School of Medicine, Faculty of Health Sciences, National and Kapodistrian University of Athens, Athens, GRC

**Keywords:** cryptorchidism, epididymal anomalies, epididymis, testicular-epididymal non-fusion, urogenital non-union

## Abstract

Complete testicular-epididymal non-fusion (cTENF) is a rare and underestimated congenital anomaly, commonly associated with an undescended testis. Dissociated epididymis is always located distally to the testis and frequently into the scrotum, resembling a testicular nubbin. We report the case of an 18-month-old boy with left cryptorchidism and cTENF initially regarded as a testicular remnant due to testicular atrophy. We highlight the distinct anatomical features of this condition to overcome the diagnostic challenge and avoid pitfalls that may lead to significant consequences.

## Introduction

Testicular-epididymal fusion anomalies (TEFA) are developmental abnormalities of the male reproductive system, affecting nearly one quarter of cryptorchid testes [1]. Complete testicular-epididymal non-fusion (cTENF) represents the rarest form, accounting for about 20% of this spectrum [1,2]. Although well documented, it is most often reported in case studies [3-8] or embedded in larger series evaluating TEFA [1,2,9]. Clinically, this anomaly represents an important intraoperative diagnostic challenge, as it may closely mimic testicular regression leading to the misinterpretation and subsequent misidentification of a non-fused testis as a remnant, potentially resulting in unnecessary orchidectomy or incomplete exploration with implications for fertility and malignancy [3,4,10]. cTENF is secondary to failure of fusion between the testis and epididymis during fetal life and shows a marked left-sided predominance [3-10]. The testis is usually normal in appearance, frequently located intra-abdominally or in a high inguinal position, and often associated with a persistent patent processus vaginalis [1,2,11]. The epididymis consistently lies distal to the testis and is frequently attached to the gubernaculum in an intrascrotal position [10]. These findings, and in particular the presence of a scrotal nubbin, may easily be misinterpreted, leading to diagnostic error and improper management [3,4,10].

We report a case of cTENF illustrating its anatomical characteristics, aiming to increase awareness and assist pediatric surgeons in managing this anatomical variation to prevent long-term implications for fertility and malignancy. To our knowledge, this is also the first report that underscores the predominantly left lateralization of complete non-fused testes in the published literature.

## Case presentation

An 18-month-old boy was referred to our department by his pediatrician with a suspected left undescended testis. Physical examination revealed a normally descended right testis, an underdeveloped left hemiscrotum, and a palpable left testis at a high inguinal position. No preoperative scrotal and inguinal ultrasound was performed, as imaging is not indicated in the presence of a palpable undescended testicle on the physical examination. The patient was scheduled for surgical exploration within the following weeks.

**Figure 1:**
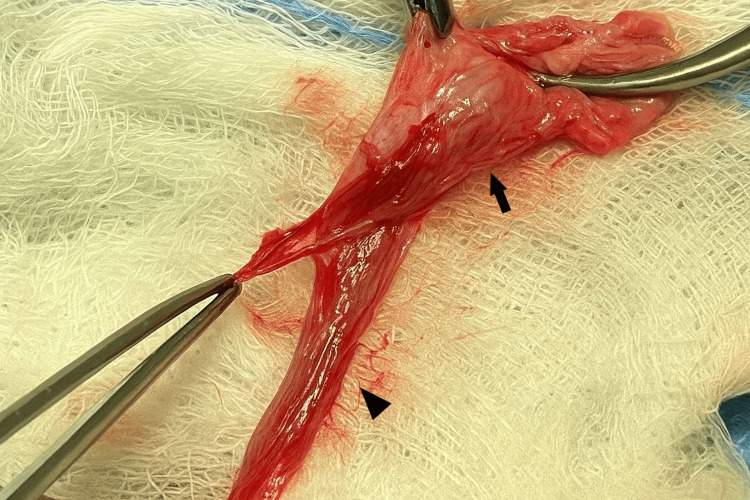
First intraoperative finding (left inguinal exploration) The vas deferens (black arrowhead) and epididymis (black arrow) surrounded by muscular layers, resembling a scrotal nubbin

**Figure 2:**
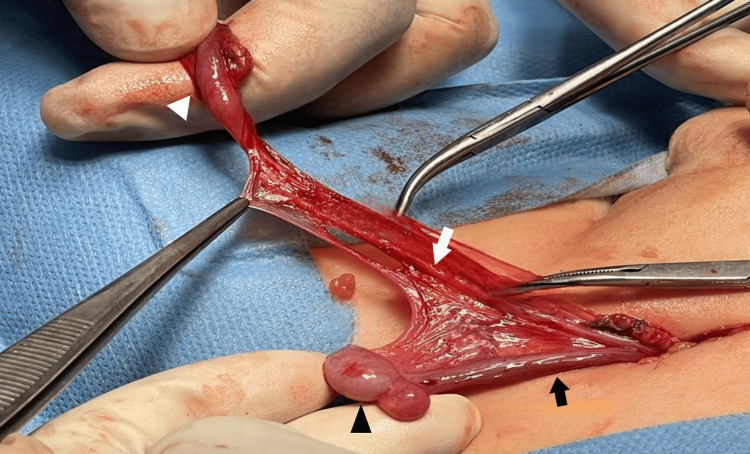
Second intraoperative finding after the opening of the left inguinal sac After further dissection and opening of the inguinal sac, the testis and the head of the epididymis (black arrowhead) were identified proximal and connected with the testicular vessels (black arrow), whereas the vas deferens (white arrow) and the epididymis (white arrowhead) lie distally to the testis with no apparent connection between them, confirming cTENF cTENF: complete testicular-epididymal non-fusion

Open left inguinal exploration was undertaken. After entering the left inguinal canal, a cord-like structure, ending in a scrotal nubbin, surrounded by a thick muscular layer was identified (Figure 1), initially interpreted as a non-viable testis. However, appreciating the clinical finding of a palpable testicle, further proximal exploration of the inguinal canal was performed, revealing a wide patent processus vaginalis, a normal-appearing left testis, and the head of the epididymis at the level of the internal ring. The gonad was supplied by its own vasculature with no evidence of vas deferens and epididymis. After this finding, the tunica layer of the then suspected nubbin was opened, and the body-tail of the epididymis was identified (Figure 2). The vas deferens and epididymis were not connected to the testis. No measurements of the distance between the testis and epididymis were recorded, as this was the first case of cTENF in our department. 

**Figure 3:**
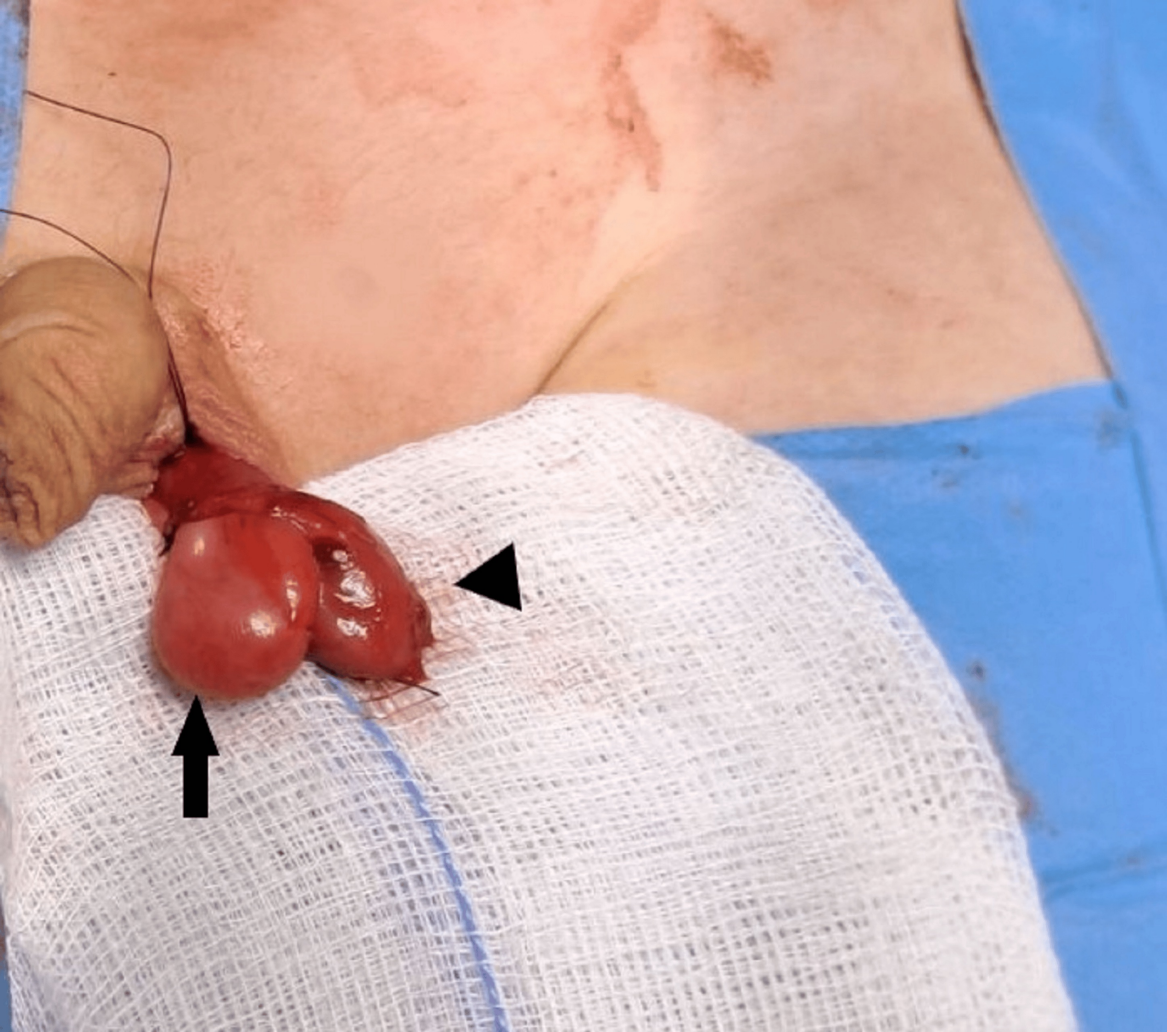
Intraoperative image after the mobilization of the left testis and the epididymis The testis (black arrow) and the epididymis (black arrowhead) after reapproximation prior to performing a tension-free subdartos orchidopexy, thus allowing the improved postoperative surveillance of both structures

Standard subdartos orchidopexy and high ligation of the inguinal sac were performed. The normally developed left testis and epididymis were reapproximated and successfully positioned without tension within the scrotum (Figure 3). No intervention on the right testicle was performed.

The postoperative course was uneventful, and the patient was discharged the same day. The parents were informed about the potential risk of impaired fertility, the need for regular follow-up examinations due to the possibility of malignancy, and the future available options to optimize fertility potential.

**Figure 4:**
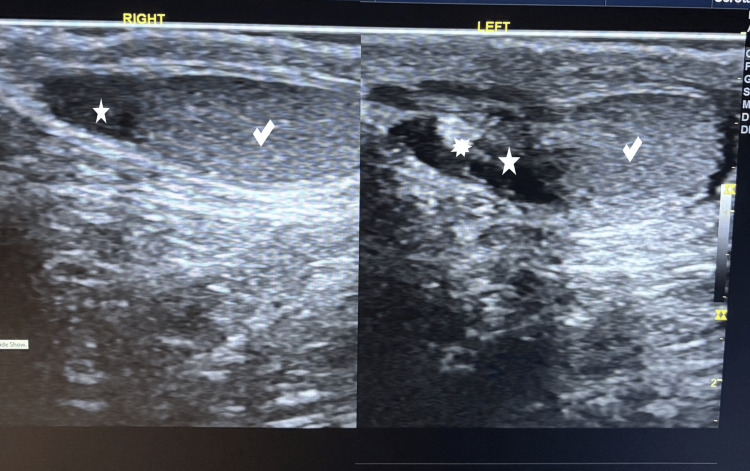
Follow-up scrotal ultrasound at the second postoperative year (age 3.5 years; longitudinal section) Longitudinal scrotal ultrasound images showing both testes (right and left check marks), with smooth contours and homogeneous echotexture of the testicular parenchyma. The right epididymis (right five-point star) appears hypoechoic and is attached to the superior pole of the testis. The left epididymis (left five-point star) demonstrates mild asymmetry with focal hyperechoic areas (multipoint star), possibly representing fibromuscular layers

**Figure 5:**
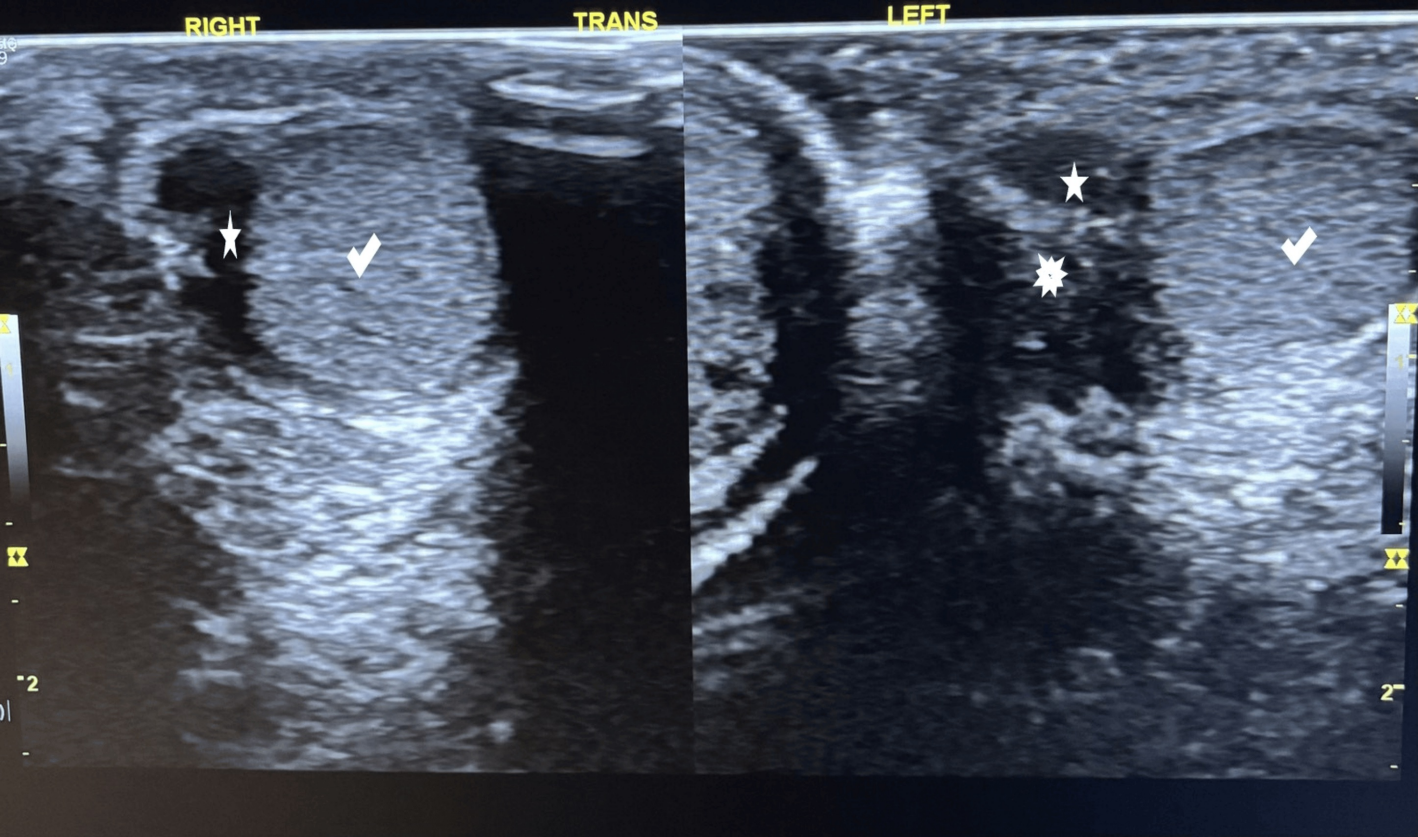
Follow-up scrotal ultrasound at the second postoperative year (age 3.5 years; transverse section) Transverse scrotal ultrasound images showing both testes (right and left check marks), in normal intrascrotal position, with smooth contours and homogeneous echotexture of the testicular parenchyma. The right epididymis (right five-point star) appears hypoechoic and is attached to the testis. The left epididymis (left five-point star) appears relatively posterior with focal hyperechoic areas (multipoint star), possibly representing fibromuscular layers

Scheduled clinical and ultrasound follow-up evaluations at one and two years postoperatively demonstrated a normally appearing and positioned testis (Figures 4-5).

## Discussion

**Figure 6:**
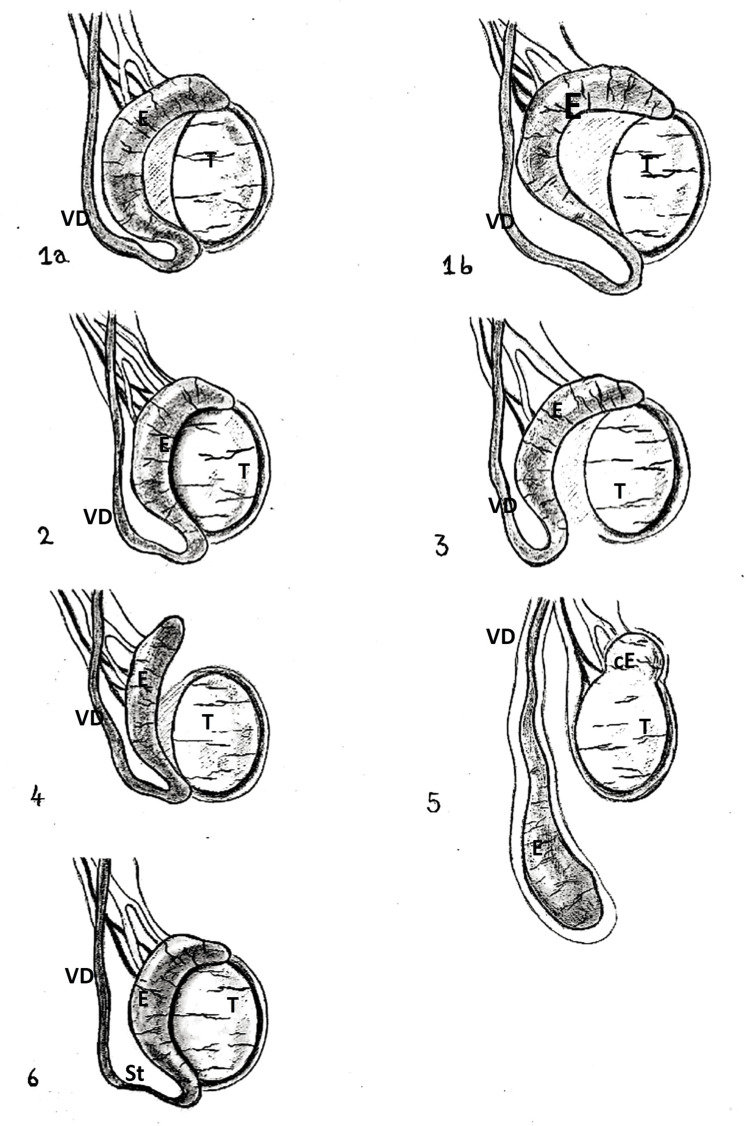
Schematic representation of the TEFA classification. 1a: Loop epididymis, in which the head and tail are attached to the upper and lower poles of the testis, respectively (normal anatomy). 1b: Long loop epididymis (normal variation). 2: The epididymis is completely attached to the testis (normal anatomy). 3: Tail non-fusion. 4: Head non-fusion. 5: Complete non-fusion. 6: Segmental epididymal atresia The figure is an original schematic illustration created by the authors, inspired by both the schematic representation and description of the TEFA classification reported by Kuçukaydin et al. [12]. Type 5 is depicted with clinical reliability, correlating with the actual anatomical configuration observed intraoperatively, where the vas deferens and epididymis are completely dissociated from the testis and caput epididymis TEFA: testicular-epididymal fusion anomalies; T: testis; E: epididymis; VD: vas deferens; cE: caput epididymis; St: stenosis

TEFA are anatomical abnormalities commonly associated with undescended testes. In a meta-analysis of English-language literature published between 1980 and 2019, including more than 4,800 cases of cryptorchidism, Qin et al. reported an incidence of TEFA in over 25% of cases [1]. Several classifications of TEFA have been developed, most of them based on a "head, tail, and complete" non-fusion system [1,12]. According to the classification by Kuçukaydin et al., six variations of testicular-epididymal fusion have been described (Figure 6). Type 1, "loop epididymis", is subdivided into type 1a, in which the epididymal head and tail are attached to the upper and lower poles of the testis, respectively, and type 1b, representing a longer epididymal loop that may extend into the inguinal canal. In type 2, the epididymis is completely attached to the testis. Types 1a, 1b, and 2 are considered normal variants, whereas types 3 (tail non-fusion) and 4 (head non-fusion) are regarded as partial fusion anomalies [1,2,12]. Types 5 and 6 represent the rarest and most severe forms, with type 5 corresponding to complete non-fusion and type 6 segmental epididymal atresia; both have been implicated in fertility impairment due to obstructive pathology [10,12].

cTENF remains an underestimated anomaly with only a few observational studies specifically addressing it [10,13,14]. In some instances, cTENF has been described simply as a fusion anomaly or even misclassified as polyorchidism, contributing to significant variability in the reported incidence [10]. In some early studies, the incidence varies between 1% and 3%, depending on the study. Kuçukaydin et al. reported an incidence of 3%, while Rachmani et al., in 880 testes, identified 14 cases (1.6%) of complete non-fusion [12,13]. In contrast, Sharma and Sen, in an observational study, reported an incidence of 8%, attributing this higher rate to meticulous surgical exploration, including the proximal tracing of the vas deferens [14]. Similarly, Qin et al., in their meta-analysis, reported an incidence of 6.3% among undescended testes [1].

cTENF is usually identified intraoperatively during inguinal exploration for cryptorchidism, though rarely may be overlooked; isolated reports describe an association with later testicular pathology, including teratoma [4,10]. Similar to our case, the vas deferens is connected to the epididymis, enveloped by a dense muscular layer [10]. The epididymis constantly lies distally to the detached testis, an anatomical arrangement documented in all reported cases and studies [3-8]. In addition, it is frequently attached to the gubernaculum within the scrotum, an association defined by Yağız et al. as "epididymo-testicular non-union with a sentinel nubbin" [10]. Among the anatomical features of cTENF, the latter closely resembles a scrotal nubbin regarded as a remnant of testicular regression, suggestive of an atrophic testis [4,10,14]. This finding is considered by some as a marker that obviates the need for further surgical exploration [14,15]. In addition, excision of any dysmorphic or hypoplastic scrotal tissue has been reported as a prudent approach [15].

Consequently, this resemblance is of considerable practical importance, as it may lead to misinterpretation and subsequent failure to identify the proximal testis. As reported by Sharma and Sen, in five out of 11 patients with cTENF with a "sentinel nubbin", the average distance between the testis and epididymis was 9.6 cm, a considerable gap that represents an additional factor contributing to overlooking the proximal testis [14].

In our case, the sentinel nubbin was the first intraoperative structure encountered (Figure 1), closely resembling an atrophic gonad. Careful reliance on the preoperative clinical findings, however, allowed the intraoperative identification of the proximal testis and prevented misdiagnosis. Similar conditions have been highlighted in previous reports, where the sentinel nubbin was occasionally misinterpreted [3,4,6]. Yağız et al. reported an incidentally detected intra-abdominal testis on ultrasonography after prior excision of an atrophic testis [10]. Papageorgiou et al. detected intra-abdominal testes on reoperation in a patient previously reported with inguinal nubbins, consistent with long-gap bilateral cTENF [4]. Furthermore, reports document intra-abdominal testes or intra-abdominal testicular tumors after surgery for presumed "vanishing testis", whereas others have demonstrated a high rate of missed cryptorchid testes among patients with intra-abdominal malignant testicular tumors, possibly attributed to missed cases of epididymo-testicular non-fusion [3,10].

In the present case, the dissociated testis was located just below the deep inguinal ring. There is a well-documented association between epididymal anomalies and high testicular position [2,10,11,13,14]. Rachmani et al. reported that nine of 14 testes with cTENF were located either intra-abdominally or sliding within the inguinal canal [13]. Yağız et al. found that three of 14 testes were intra-abdominal, while 11 were near the internal inguinal ring [10]. Similarly, Sharma and Sen reported intra-abdominal testes in six of 11 cases of complete non-fused testes [14]. This feature has been described consistently in most case reports [3,4,6].

Cinislioglu et al., in a cohort of 241 cases, demonstrated that both the incidence and severity of epididymal anomalies significantly decreased as the position of the testis descended from intra-abdominal to the internal inguinal ring, external inguinal ring, and distal external inguinal ring (100%, 54.2%, 26.4%, and 19.1%, respectively) [11]. Qin et al., in their analysis, reported an overall prevalence of TEFA of 71.1% among intra-abdominal testes and 32.1% among inguinal testes, indicating that intra-abdominal testes are more likely to exhibit epididymal anomalies [1].

Another anatomical feature, strongly associated with TEFA and well documented in several studies, is the presence of a patent processus vaginalis [1-3,5,11]. Reported incidences vary, with some series describing up to 75% association [14]. Cinislioglu et al. found that the incidence of epididymal anomalies in cases with patent processus vaginalis was significantly higher than in those with an obliterated processus vaginalis (50% vs. 12.1%) [11]. Similar findings were reported by Caterino et al., with more than 50% association [2]. Yağız et al., in their study of 838 patients including 14 cases of cTENF, reported the presence of a patent processus vaginalis or inguinal sac in all cTENF cases, a finding also confirmed in the majority of case reports [3,5,10]. Logsdon et al., in a series of 110 testes from 87 patients, observed that seven of 10 cases of left-sided cTENF were associated with a patent processus vaginalis, whereas in the same cohort, three right-sided cases were found with obliterated processus vaginalis, even in the same patient [9].

In our case, the dissociated testis was located just below the deep inguinal ring, with a patent processus vaginalis, further supporting the notion that high testicular position and persistence of the processus vaginalis are closely related to epididymal non-fusion.

Another notable aspect of cTENF is its predilection for left lateralization, a phenomenon that, although reported, remains underemphasized and poorly studied. Whereas TEFA and cryptorchidism are typically right-sided and large studies found no association of laterality with TEFA [1,2,16], more complex urogenital anomalies, including cTENF, have been predominantly reported on the left [16-18]. In all published case reports of cTENF, the anomaly has been described on the left side [3-8]. Yağız et al. reported a 79% incidence among 14 cases, and Logsdon et al. identified 10 cases of cTENF among 110 testes, of which seven were left-sided, two were bilateral, and one was right-sided [9,10]. Abdelmohsen et al. reported left lateralization in 72% of cryptorchid patients with epididymal anomalies, and Honoré described nine of 11 unilateral congenital anorchias on the left [17,18]. Kirkpatrick et al., in an analysis of 1455 pediatric cases of impalpable undescended testis, estimated that 59% of cases and 61% of Wolffian duct abnormalities, particularly unilateral absence of the vas deferens, were left-sided [16].

The process of testicular-epididymal fusion and descent is complex [2,19]. The testis and epididymal head arise from the gonadal ridge, whereas the epididymal body and tail and the vas deferens derive from the mesonephric duct and tubules [2]. Fusion is achieved by canalization of the rete testis and mesonephric tubules by the 24th gestational week [2,4]. This process is influenced by hormonal and mechanical factors, including androgens, vascular development, intra-abdominal pressure, and the epididymis itself, which, by remaining anatomically distal to the testis throughout development, plays an essential role in descent; failure of fusion may consequently prevent testicular migration [2,10,13]. According to Rachmani et al., only complete non-fusion reliably impairs testicular descent, whereas partial or isolated defects may still permit epididymo-testicular union and migration [13]. Kraft et al. emphasized that decreased androgen production alone is insufficient to explain cTENF; therefore, vascular development may play a pivotal role in both failure and the anatomical features of this anomaly [17,19].

In early stages, both the gonad and mesonephric structures are supplied by the lateral branches of the dorsal aorta; later, this supply diverges into the internal spermatic artery for the testis and branches of the internal iliac artery for the vas deferens [17]. Some authors have suggested that transient ischemic events during this vascular transition may lead to the segmental disruption of the epididymis continuity [17,18]. Venous drainage is likewise asymmetric: the right spermatic vein drains directly into the inferior vena cava, whereas the left drains into the renal vein. This configuration predisposes the left side to venous stasis and obstruction, a vulnerability that may be accentuated by a mobile kidney, causing the kinking of the left spermatic vein [18]. As reported by Honoré, during early intrauterine development, before venous anastomoses across the midline are established, such obstruction could precipitate ischemia, atrophy, and fibrosis of the testis and epididymis, thereby preventing normal descent [18]. Although originally proposed to explain the predominance of left-sided unilateral anorchia, we believe that this mechanism, although hypothetical, may provide a coherent embryological and vascular framework, supported by the highly conserved human left-right asymmetry early in embryogenesis, for understanding both the failure of testicular epididymis fusion and the left predominance in cTENF [16,17].

Once the anomaly was identified, we performed a subdartos scrotal orchidopexy, reapproximating the two structures in order to allow regular surveillance and preserve the potential for future reconstructive procedures. A structured follow-up with ultrasound and clinical evaluation was recommended to the parents.

The management of cTENF, in the absence of established guidelines, remains controversial and largely depends on the surgeon's preference [3-8,10]. Reported management approaches range from orchidopexy with nubbin preservation to orchidopexy with nubbin excision [3,5,6] or, in selected cases when orchidopexy is not feasible, excision of both the testis and the nubbin [10]. Some advocate the excision of the highly located viable testis for the assumed higher malignancy rate in a testis with no drainage and thus no contribution to fertility [9,10,14]. In contrast, studies report that cTENF is not correlated with significant histological gonadal abnormalities [4,19], while others suggest that non-fusion anomalies of the epididymis represent forms of developmental delay and should not be considered a congenital dysplastic organ [13]. This is further supported by accounting that 69% of the testes with severe anomalies of ductal fusion showed the preservation of germ cells, indicating that the testis may be hormonally active and contributing to the child's development [10,14].

These findings support a gonad-preserving approach, a strategy consistent with most published series or case reports [3-5,10,14]. Orchidopexy, when feasible, is considered by most as the option of choice [5,10]. In an observational series, orchidopexy was feasible in 13 out of 14, with orchidectomy required in only one case due to the presence of a very thin and short vascular pedicle. The scrotal nubbin was excised in only four patients while left in the scrotum in the rest of the 10 patients, at the discretion of the attending surgeon [10]. Similarly, Sharma and Sen reported successful orchidopexy in eight out of 11 patients, and comparable approaches have been described in most published case reports [14].

Fixation of the contralateral descended testicle, in case of cTENF, is not studied. Cases of isolated epididymal torsion associated with cTENF, particularly when the epididymis is widely separated, have been reported [14]. In contrast, in a large cohort study, not a single contralateral descended testis with cTENF was identified [13]. Consequently, there is no consensus in the literature regarding the routine fixation of a solitary testis when there are no predisposing factors increasing the risk of torsion [20]. Current scientific evidence does not clearly demonstrate whether contralateral fixation is necessary; therefore, the decision should be individualized according to the clinical scenario and the surgeon's preference [20]. However, if contralateral orchidopexy is undertaken, the dartos pouch sutureless technique is considered the preferred approach [20].

Full documentation of the operative findings and appropriate counseling regarding fertility and malignancy risk are recommended [10,14]. Parents should be informed about the necessity of regular clinical and imaging surveillance of the testis and the option of contralateral testicular fixation. In addition, counseling about available fertility preservation options and ongoing reproductive technology developments may assist long‑term planning [10,14].

## Conclusions

cTENF represents a rare and underestimated yet clinically significant anomaly that poses important diagnostic challenges, particularly due to its frequent resemblance to a testicular nubbin. Pediatric surgeons should be aware of its characteristic features, as accurate identification is crucial to prevent misinterpretation and avoid misdiagnosis and serious malignant complications. Diagnostic pitfalls, repeatedly described in the literature, highlight the importance of meticulous intraoperative assessment, with proximal inguinal or even laparoscopic exploration when a clinical scenario involves a left-sided scrotal nubbin, particularly in the presence of a patent processus vaginalis.

Orchidopexy, whenever feasible, is regarded as the treatment of choice, given the generally normal histological profile of the gonad. Further anatomical and experimental research is warranted to investigate the apparent left-sided predominance of the anomaly, to define its true incidence, and to establish evidence-based management strategies.
